# Correlations of Clinical and Laboratory Characteristics of COVID-19: A Systematic Review and Meta-Analysis

**DOI:** 10.3390/ijerph17145026

**Published:** 2020-07-13

**Authors:** Ramy Abou Ghayda, Jinhee Lee, Jun Young Lee, Da Kyung Kim, Keum Hwa Lee, Sung Hwi Hong, Young Joo Han, Jae Seok Kim, Jae Won Yang, Andreas Kronbichler, Lee Smith, Ai Koyanagi, Louis Jacob, Jae Il Shin

**Affiliations:** 1 Division of Urology, Brigham and Women’s Hospital and Harvard Medical School, Boston, MA 02115, USA; ramy.aboughayda@gmail.com; 2 Department of Global Health and Population, Harvard T.H. Chan School of Public Health, Boston, MA 02115, USA; sunghwihong@gmail.com; 3 Department of Psychiatry, Yonsei University Wonju College of Medicine, Ilsan-ro 20, Wonju 26426, Korea; jinh.lee95@yonsei.ac.kr; 4 Department of Nephrology, Yonsei University Wonju College of Medicine, Ilsan-ro 20, Wonju 26426, Korea; junyoung07@yonsei.ac.kr (J.Y.L.); ripplesong@yonsei.ac.kr (J.S.K.); kidney74@yonsei.ac.kr (J.W.Y.); 5 Yonsei University Wonju College of Medicine, Ilsan-ro 20, Wonju 26426, Korea; judi_kim@naver.com; 6 Department of Pediatrics, Yonsei University College of Medicine, Yonsei-ro 50, Seodaemun-gu, Seoul 03722, Korea; AZSAGM@yuhs.ac; 7 Department of Pediatrics, Samsung Changwon Hospital, Sungkyunkwan University School of Medicine, Changwon 51353, Korea; 82agnes@hanmail.net; 8 Department of Internal Medicine IV, Nephrology and Hypertension, Medical University Innsbruck, Innsbruck 6020, Austria; andreas.kronbichler@i-med.ac.at; 9 The Cambridge Centre for Sport and Exercise Sciences, Anglia Ruskin University, Cambridge CB1 1PT, UK; Lee.Smith@anglia.ac.uk; 10 Parc Sanitari Sant Joan de Déu/CIBERSAM, Universitat de Barcelona, Fundació Sant Joan de Déu, Sant Boi de Llobregat, 08830 Barcelona, Spain; a.koyanagi@pssjd.org (A.K.); louis.jacob.contacts@gmail.com (L.J.); 11 ICREA, Pg, Lluis Companys 23, 08010 Barcelona, Spain; 12 Faculty of Medicine, University of Versailles Saint-Quentin-en-Yvelines, 78180 Montigny-le-Bretonneux, France

**Keywords:** COVID-19, correlation, clinical characteristics, laboratory findings, treatment

## Abstract

(1) Background: The global threat of Coronavirus disease 2019 (COVID-19) continues. The diversity of clinical characteristics and progress are reported in many countries as the duration of the pandemic is prolonged. We aimed to perform a novel systematic review and meta-analysis focusing on findings about correlations between clinical characteristics and laboratory features of patients with COVID-19. (2) Methods: We analyzed cases of COVID-19 in different countries by searching PubMed, Embase, Web of Science databases and Google Scholar, from the early stage of the outbreak to late March. Clinical characteristics, laboratory findings, and treatment strategies were retrospectively reviewed for the analysis. (3) Results: Thirty-seven (*n* = 5196 participants) COVID-19-related studies were eligible for this systematic review and meta-analysis. Fever, cough and fatigue/myalgia were the most common symptoms of COVID-19, followed by some gastrointestinal symptoms which are also reported frequently. Laboratory markers of inflammation and infection including C-reactive protein (CRP) (65% (95% confidence interval (CI) 56–81%)) were elevated, while lymphocyte counts were decreased (63% (95% CI 47–78%)). Meta-analysis of treatment approaches indicated that three modalities of treatment were predominantly used in the majority of patients with a similar prevalence, including antiviral agents (79%), antibiotics (78%), and oxygen therapy (77%). Age was negatively correlated with number of lymphocytes, but positively correlated with dyspnea, number of white blood cells, neutrophils, and D-dimer. Chills had been proved to be positively correlated with chest tightness, lung abnormalities on computed tomography (CT) scans, neutrophil/lymphocyte/platelets count, D-dimer and CRP, cough was positively correlated with sputum production, and pulmonary abnormalities were positively correlated with CRP. White blood cell (WBC) count was also positively correlated with platelet counts, dyspnea, and neutrophil counts with the respective correlations of 0.668, 0.728, and 0.696. (4) Conclusions: This paper is the first systematic review and meta-analysis to reveal the relationship between various variables of clinical characteristics, symptoms and laboratory results with the largest number of papers and patients until now. In elderly patients, laboratory and clinical characteristics indicate a more severe disease course. Moreover, treatments such as antiviral agents, antibiotics, and oxygen therapy which are used in over three quarters of patients are also analyzed. The results will provide “evidence-based hope” on how to manage this unanticipated and overwhelming pandemic.

## 1. Introduction

The cause of an outbreak of an unidentified respiratory disease, first detected in Wuhan, China, has been identified as a type of novel sub-coronavirus, initially named 2019-novel coronavirus (2019-nCoV) [1]. The World Health Organization (WHO) has officially named the virus “severe acute respiratory syndrome coronavirus 2 (SARS-CoV-2)”, and the associated disease “coronavirus disease 2019 (COVID-19)” [2]. Since February 2020, COVID-19 cases of infection have rapidly expanded at a global level, and as of March 2020, more than 118,000 cases have been reported across 114 countries. On the 11 March 2020, WHO acknowledged that the virus would likely spread to all countries across the globe and declared the coronavirus outbreak a pandemic [3]. This is the fifth pandemic caused by the emergence of a novel virus since the 20th century, following the Spanish Influenza in 1918–19 (an estimated 20–50 million deaths globally), the Asian Influenza in 1957–58 (1–4 million deaths globally), the Hong Kong Influenza in 1968–69 (1–4 million deaths worldwide), and the 2009 pandemic caused by A (H1N1) (100,000–400,000 deaths worldwide) [4].

COVID-19 is thought to be primarily transmitted via human-to-human transmission pathways, through mediators such as droplets, contact, and fecal-oral transmission [5,6]. Several coronaviruses are known to cause respiratory, gastrointestinal (GI), liver, and neurologic diseases in animals; however, only seven are known to cause disease in humans [7]. Three of these seven coronaviruses can cause much more severe, and sometimes fatal respiratory infections in humans when compared to other coronaviruses and have caused major outbreaks of deadly pneumonia in the 21st century. These coronaviruses include severe acute respiratory syndrome (SARS), Middle East Respiratory Syndrome (MERS), and COVID-19, all found to be enveloped RNA viruses. COVID-19-infected cases continue to increase, with many cases being unreported, and so the lethality and infectivity of COVID-19 remain unknown. To date, more than 344,000 people have lost their lives to COVID-19, which is six times the total number of deaths reported at the end of the SARS outbreak in 2002 [8], and 12 times the estimated number of deaths caused by MERS in 2012 [9].

The WHO described the symptoms of 55,924 laboratory confirmed COVID-19 cases in China in the period up to February 2020 [10]. According to the report, many of the symptoms are shared with those of the common flu or cold, the most common symptoms being fever and a dry cough. Following this report, many more studies have been carried out by multiple governments and researchers to assess the clinical phenotype and diagnostic findings of COVID-19. Some of these studies display high methodological quality and include a relatively large number of cases. However, most of these studies have been conducted with heterogeneous study design and insufficient sample size, resulting in a failure to draw consistent conclusions. Therefore, in the present study, we aimed to perform a systematic review and meta-analysis to find out correlations among clinical characteristics, laboratory findings and treatment strategies, for the first time, including all studies published so far.

## 2. Materials and Methods 

### 2.1. Search Strategy and Data Extraction

In order to identify studies relating to the clinical characteristics, laboratory findings and treatments of COVID-19 in different countries, we carried out a search of PubMed, Embase, and Web of Science databases. A search of Google Scholar was also conducted using the same keywords to identify any additional relevant articles. As reports are being updated every day, a rapid review was conducted to summarize the current information on COVID-19, including all articles available without language limitations.

The search terms used included: “2019-nCoV”, “novel coronavirus”, “NCP”, “COVID-2019”, “COVID-19”, and “SARS-CoV-2”. We also narrowed the searching scope by including the terms: “clinical”, “characteristics”, “features”, and “findings”. The search time was limited to “2019–2020”. The inclusion criteria included studies focusing on clinical characteristics and symptoms, radiological examination results, and laboratory examination results of patients with SARS-CoV-2 infection. We excluded articles that had been published repeatedly or withdrawn, did not include the research indicators needed for meta-analysis, had five or fewer participants, or were single case reports.

Three authors (J.I. Shin, J. Lee and D.K. Kim) independently searched for articles and extracted data from the identified studies. The titles and abstracts of the literature were first screened to exclude articles that did not meet the inclusion criteria. Following this, the remaining articles were reviewed in full to decide which studies were appropriate to include in the final analysis. If any disagreement between authors occurred, the full text and extracted data were reviewed by other coauthors to verify accuracy.

### 2.2. Statistical Analysis

For reports on clinical characteristics, studies were included in the meta-analysis if they reported the number of cases and sample denominator results using a 95% confidence interval (CI), and categorical variables expressed as *n* (%) and *n*/N (%). For reports on laboratory findings, we collected the continuous outcomes, and the mean difference with 95% CI. When studies reported continuous variables as median and range or interquartile range, we estimated the mean and standard deviation (SD) using the method described by Wan, et al. and the calculator these authors have provided [11]. R version 3.33 (The R Foundation for Statistical Computing, Vienna, Austria) was used for all meta-analyses [12]. The metapackage was used to generate forest plots, pooled estimates, and to evaluate the potential of publication bias.

## 3. Results

### 3.1. Summary of Previously Published Meta-Analyses

Until now, a total of eight previous published papers on COVID-19 related to clinical symptoms and laboratory findings have been identified. Table 1 shows the findings of each paper. First, two papers—Rodriguez-Morales et al. [13] and Li et al. [14]—were analyzed with data from 19 and 10 papers, respectively, and clinical symptoms, laboratory findings and outcome of patients were summarized. However, the number of papers and patients collected initially were too small and correlation between each variable was not investigated. Zhang et al. [15] and Zeng et al. [16] have conducted meta-analyses with only laboratory findings of COVID-19 patients in 4663 and 3962 patients, respectively. These papers did not describe clinical features, but only summarized laboratory findings. Wang et al. [17] summarized the clinical characteristics and laboratory findings in children but it is difficult to apply these to all adults and correlation between the findings is not investigated. Lastly, Lovato et al. [18], Fu et al. [19] and Li et al. [20] analyzed clinical characteristics and laboratory findings associated with critical outcomes such as intensive care unit (ICU) care or case-fatality rate (CFR). These cases also did not describe the correlations between clinical and laboratory characteristics.

### 3.2. Study Selection and General Characteristics

Thirty-six eligible publications were finally selected and assessed for full text after a selection process. The analysis was restricted to clinical characteristics, laboratory findings including imaging, and treatment, leading to inclusion of 31, 24, and 10 papers, respectively. Detailed information of the studies is presented in Supplementary Table S1. Thirty-three papers analyzed Chinese cases, while three papers reported cases from outside of China. The follow-up of patients ranged from a minimum of nine days to a maximum of 52 days. All studies were retrospective in nature: 34 studies were cross-sectional studies, and two were case e-series studies. Specifically, two studies targeted children, and one study targeted pregnant women. Our review included 5196 COVID-19 patients. All the included studies were published in 2020.

### 3.3. Clinical Characteristics

**Figure 1:**
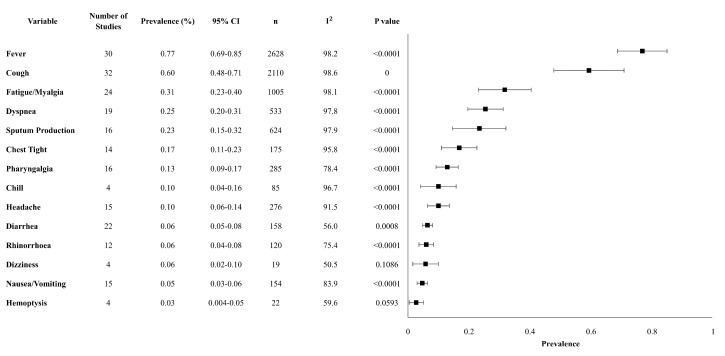
Forest plot of clinical characteristics. Abbreviations: CI: confidence interval, *n*: number, I^2^: heterogeneity.

Our review analyzed 14 clinical characteristics. The most prevalent clinical characteristics were fever and cough. Fever occurred in 76.8% (95% CI 68.7–84.9%), cough occurred in 59.3% (47.8–70.8%), and fatigue/myalgia occurred in 31.7% (23.1–40.4%) of patients. Upper and lower respiratory symptoms were reported less frequently, with dyspnea in 25%, sputum production in 23%, chest tightness in 17%, pharyngalgia in 13%, and rhinorrhea in 6% of patients. Gastrointestinal symptoms, including diarrhea and nausea/vomiting occurred in 6% and 5% of patients, respectively. The above-mentioned prevalence was statistically significant, with a *p*-value < 0.01 (Table 2). The overall meta-analysis results of the clinical characteristics are shown in Figure 1.

### 3.4. Laboratory Findings and Chest Imaging

**Figure 2:**
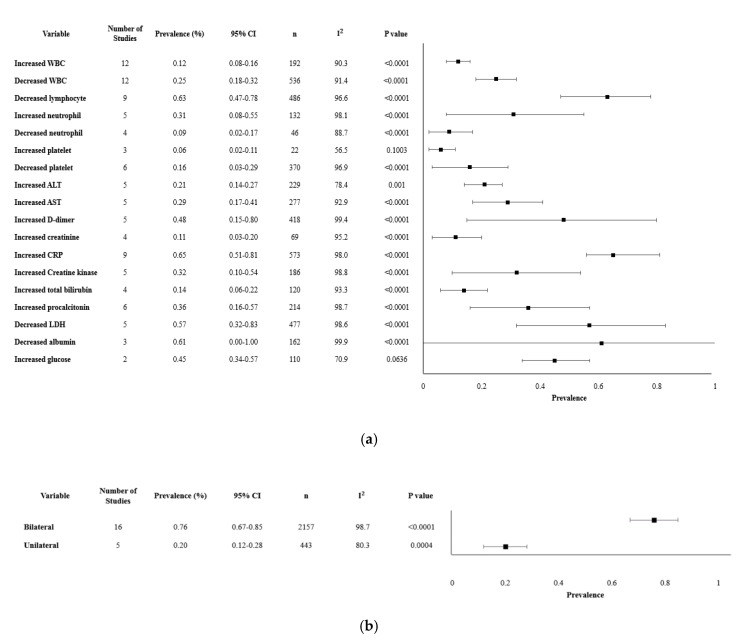
Forest plot of laboratory parameters and chest imaging. (**a**). Forest plot of laboratory findings. (**b**). Forest plot of chest imaging findings.

Our review analyzed about 18 laboratory findings and 2 chest imaging. The most significant findings were lymphocytopenia (62.7%, 95% CI 47.1–78.3%), decreased albumin (60.6%, 95% CI 0–100%) and increased lactate dehydrogenase (LDH) (57.4%, 95% CI 31.7–83.1%). Other clinically meaningful changes observed were an increase in liver function enzymes, including alanine aminotransferase (ALT), aspartate aminotransferase (AST), and total bilirubin, with altered levels reported in 28.9% (95% CI 23.9–33.9), 34.2% (95% CI 28.6–39.7) and 10.7% (95% CI 6.6–14.8) of the cases. CRP, a marker of inflammation, was significantly elevated with mean levels of 22.8 mg/L (95% CI 16.1–28.2). Specific laboratories and their variability, reported as an increase or decrease in the values, are presented in Table 2. Imaging methods such as chest computed tomography (CT) and X-rays revealed that 75.8% (95% CI 66.9–84.8%) had bilateral lung involvement, while 19.9% (95% CI 11.7–28.0%) had only unilateral findings (Table 2). The overall meta-analysis results of the laboratory findings and imaging are shown in Figure 2.

### 3.5. Treatments

**Figure 3:**
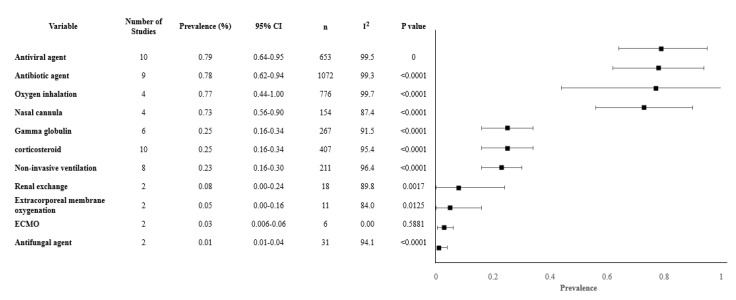
Forest plot of treatments. Abbreviations: CI: confidence interval, *n*: number, I^2^: heterogeneity.

Our review analyzed 11 different treatments modalities and revealed interesting findings. The most common treatment approaches had a similar range of prevalence and included antiviral measures (79.4%, 95% CI 63.6–95.2%), antibiotics (77.7%, 61.5–93.9%), and oxygen inhalation (77%, 95% CI 43.9–100%). Other treatment modalities such as gamma-globulin, and the use of corticosteroids were less common, used in less than a third of the patients. Other treatments for severe cases that were observed in less than 10% included non-invasive ventilation, renal replacement therapy, and extracorporeal membrane oxygenation. (Table 3). The overall meta-analysis results of the treatments modalities are shown in Figure 3.

### 3.6. Correlation of Clinical Characteristics with Demographics and Laboratory Findings

Until the writing of this report, up to the best of our knowledge, this is the first time that COVID-19 symptoms were found to be correlated with each other (Table 4). Chills had the highest number of perfect positive correlations. We found a perfect correlation coefficient of 1 with a *p*-value of 0.01 statistical significance with chest tightness, radiographic lung abnormalities, neutrophil, lymphocyte, platelet counts, D-dimer, and CRP. On the contrary, chills were strongly negatively correlated with age, with a correlation coefficient of −0.963. Lymphocytes had a significant negative correlation with age with a Pearson correlation (PC) of −0.651, statistically significant at a *p*-value of 0.01. Dyspnea had the strongest positive correlation with age, with a correlation coefficient of 0.764 (*p* = 0.001). Cough was highly correlated with sputum production with a positive correlation of 0.674 (*p* = 0.01). Lung abnormalities had a strong positive correlation with an elevation of CRP, with a PC of 0.725. A selective correlation demonstrated that age was positively correlated with WBC count, dyspnea, and neutrophil counts. Sputum production was positively correlated with cough (correlation of 0.674) and headache with a correlation of 0.759. Finally, WBC was also positively correlated with platelet counts, dyspnea, and neutrophil counts with the respective correlation of 0.668, 0.728, and 0.696.

## 4. Discussion

Up until the date of writing this report, almost all countries globally are still struggling with the COVID-19 pandemic. Regardless of race, geography, global power, level of development, and advancement, the virus is sparing almost no place on the planet [1,21,22]. The medical community, grassroots groups, for-profit, as well as non-profit organizations, governmental establishments, and policymakers are struggling collectively [23]. They are coming up with measures, recommendations, and obligations in the hope of mitigating the morbidity and mortality of the disease and limiting its spread as much as realistically as feasible [6]. Most of these stakeholders are building their suggestions and proposals based on the scientific evidence that has been accumulating since the virus was first identified in December 2019 in Wuhan, China [24].

The understanding of COVID-19 is a continuously evolving path. Risk factors and susceptibilities to the virus, vulnerabilities of patients, response to treatment are cumulatively unfolding as the pandemic is spreading [25,26,27]. The knowledge and assertion of the virus manifestations are unrevealing themselves with every single case identified. Therefore, the management and measures for the prevention and treatment of COVID-19 are mostly based on the synthesis of evidence that is provided by the scientific community [28,29]. Our systematic review and meta-analysis provide additional data that can be relied on in the aspiration to optimize the management of the COVID-19 pandemic and provide hope of better survival and outcomes for millions around the world. The knowledge of this disease is mainly provided by the sum of its clinical signs and symptoms, imaging hallmarks and laboratory findings. COVID-19 represents a full spectrum ranging from asymptomatic conditions on one end of the spectrum, to a fatal disease at the other end.

As mentioned in Table 1, there have been eight meta-analyses about clinical characteristics and laboratory findings of COVID-19 published so far. However, two of these articles [13,14] only included papers published until February, so the number of included papers and patients was too small and the relationship between each variable was not analyzed. Other two meta-analyses [15,16] were only for laboratory findings. Including all four of these articles, there has been no content of correlation between variables and no treatment was mentioned.

In this meta-analysis, we analyzed the most recent and the highest number of papers and patients with COVID-19. This meta-analysis of 36 studies included one of the largest sample sizes of 5196 patients with COVID-19 and contained high literature quality and convincing evidence with comprehensive analysis, which was statistically significant. Our results showed that the main clinical manifestation of the infection included fever and cough, in 77% and 60% of patients, respectively. This is in accordance with the early reports coming from China. All the studies regarding novel coronavirus, report fever and cough as the most common, rendering them pathognomonic signs and symptoms of the infection. One of the first sets of data on the clinical features of Chinese patients with SARS-CoV-2 reported fever in 98% of infected patients, 44% having fever between 38.1 °C and 39 °C and 34% above 39.0 °C. Cough was present in 76% of their studied population [1]. Subsequent studies including a bigger sample size showed clinical manifestations in ranges similar to those we reported in our meta-analysis. Compared to SARS and MERS, the frequency of fever as a leading symptom was comparable in COVID-19 patients, however, the frequency of cough was higher than reported in MERS patients [30,31]. Other signs and symptoms such as myalgia/fatigue (31%), dyspnea (25%), sputum production (23%) and chest tightness (17%) have also been associated with COVID-19 [27,28,32,33].

Our meta-analysis revealed GI symptoms such as nausea/vomiting and diarrhea to be reported in 5 and 6% respectively, significantly less prevalent than respiratory symptoms. GI symptoms in patients with COVID-19 have been initially overlooked and not associated with the infection. More recent meta-analysis dedicated specifically to GI manifestation among COVID-19 infected individuals reported a much higher prevalence. The most recent data published in April 2020, reported that the pooled prevalence of GI manifestations was as high as 18% [13]. The most common symptoms were anorexia in 27% of patients, followed by diarrhea 12%, nausea/vomiting in 10% [13]. GI symptoms were less likely to occur in COVID-19 compared to SARS and MERS, occurring on average in 5–7% versus 20–25% of cases [25,34].

The most pathognomonic laboratory finding from our data was a decrease in lymphocytes in 63% of cases (95% CI 47–78%). It has been well established that COVID-19 is associated with immune system dysregulation [35,36]. The most common findings were, similar to our results, increase in neutrophil–lymphocyte ratio and T lymphopenia, with a decrease in CD4+ T cells and subsequent damage to T lymphocytes [35]. In addition to the quantitative dysregulation, qualitative abnormalities in the immune system have been associated with SARS-CoV-2-positive individuals [35]. The function of natural killer cells and CD8+ T cells was exhausted among these patients [35]. It was even argued that the degree of lymphopenia can predict disease severity, progression and prognosis of COVID-19 [37]. The decrease in lymphocytes might be secondary to the overwhelming and subsequently devastating immune reaction secondary to excessive T lymphocyte activation. One potential explanation is that viral infection might induce a relative increase in Th17 cells and high CD8+T cells leading ultimately to their depletion [38]. Other theories include the presence of virus ACE2 receptors in abundance on the lymphocytes which make them susceptible to early destruction [39], the direct destruction of the lymph organs and that the induced pro-inflammatory cytokines (i.e., IL-6, TNF-α) lead to the destruction of the lymphocytes [38,40]. This observed lymphopenia was similarly observed in SARS-infected patients [41].

In this meta-analysis, we found that two-thirds of the patients have an elevation of CRP. In COVID-19, CRP correlated with lung lesions, the severity of pneumonia and overall disease severity in the early stage of the disease [42,43]. Liver function enzymes including ALT and AST were slightly increased in around 30% of analyzed patients. This is in accordance with the most recently reported literature [44,45]. The underlying pathophysiologic process include viral hepatitis, secondary to the overwhelming immune reaction, or microvascular injury driven by a sepsis-like picture [45,46,47,48]. Elevation of liver function enzymes among some patients might also be attributed to overexpression of the virus ACE2 receptors [48]. Regardless of the etiology, the liver derangements are thought to be mild and have been considered as a collateral damage of the virus [44]. Albumin on the other hand is decreased, as has been reported in 61% of analyzed cases. These results are in accordance with findings during the SARS epidemic [1,49].

We only analyzed imaging studies with relation to lung injury. Among patients undergoing imaging techniques, 76% of the patients in the analysis showed bilateral ground glass appearances. On the other hand, only 20% showed these lesions unilaterally. Our results indicate lower frequencies in comparison to other studies. Bilateral pulmonary involvement in patients with COVID-19 reached frequencies as high as 98% [50]. Typical findings included consolidation, lobular and sub-segmental, or ground-glass opacities in less severe cases such as nodular and patchy shadowing and pleural effusion. In patients with severe disease, almost all patients universally turned into “white lung” [50]. However, most studies argue that the radiologic manifestations of COVID-19 can show a diverse pattern, with the process involving both the lung parenchyma and the interstitium [43].

Our meta-analysis of treatment approaches revealed three predominant modalities, antiviral agents (79%), antibiotics (78%), and oxygen therapy (77%). This is in discordance with other meta-analysis performed at an earlier stage. Others have shown that the majority of patients received antibiotics, while other therapy modalities were less prevalent [23]. Up until the writing of this manuscript, no single validated guideline for treatment of COVID-19 has been issued by any medical or scientific entity. Randomized control trials comparing efficacy and safety of treatment have been initiated but standardization of such trials is difficult (i.e., patient selection due to severity, co-morbidities, age, etc.). For example, the use of corticosteroids as a potent anti-inflammatory drug has been the subject of many medical debates. Although the WHO recommended against its routine usage [51], multiple meta-analyses have shown that medical groups and hospitals are using it as part of their internal virus management policies [1,23,32]. The same has been observed with other treatment modalities, such as anti-viral agents. Although many reports have shown promising response rates and clinical improvement [52,53,54], a standardized and validated protocol using antivirals as a treatment for COVID-19-infected patients is still nonexistent, and most dosages and durations are used on a compassionate basis. In our meta-analysis, other management modalities (e.g., gamma-globulin and corticosteroids) were less likely to be used and were initiated in less than 25% of patients.

Our study is the first to perform correlations between symptoms, characteristics, and laboratory values of SARS-CoV-2-infected patients. Chills had the greatest number of perfect positive correlations, i.e., with chest tightness, lung abnormalities on CT scans, neutrophil/lymphocyte/platelet count, D-dimer and CRP, and a strong negative correlation with age and lymphocyte count, both factors known to be associated with mortality. Advanced age of the infected patients correlated with the degree of lymphopenia, which again provides evidence that the elderly exhibit a reduced immunocompetence. On the contrary, dyspnea, again a predictor of mortality, had the strongest positive correlation with age. As expected, cough was positively correlated with sputum production. Finally, pulmonary abnormalities had a strong positive correlation with elevation of CRP, which is indicative of disease progression. Our findings are in accordance with all the existing scientific literature up until now. The calculated Pearson correlation showed once again the strong and clinically significant correlation between elevated CRP and lung lesions, and the association between the severity of the disease/patients age and lymphopenia. We found that chills had the strongest association with multiple variables and manifestations of the disease. Chills have been overlooked in previous studies, and we feel that it warrants future attention.

Our report is the first to identify potential correlations between symptoms, laboratory, radiologic, and clinical characteristics. Our data has once again provided evidence for the role of CRP and lymphopenia, among other markers in the stratification of the disease’s severity and prognosis. It has also provided new insight regarding chills, and its perfect positive correlation with other signs and symptoms of the disease, and the role age plays in severity of the disease through its positive correlation with symptoms such as dyspnea and WBC. Additionally, we have provided the most up to date and one of the most comprehensive meta-analyses of clinical manifestations, laboratory and imaging findings and treatment strategies.

Our meta-analysis has some limitations. First, most of the data analyzed originated from China, except for three studies. Second, bias in results reporting could not be appropriately assessed because of the novelty of the disease and the relative paucity of existing evidence. Third, no papers were excluded based on publication bias, because our study aimed at being as comprehensive as possible including all the current data. In addition, newer treatment approaches developed were not reflected because the research has not accumulated until now. Furthermore, all studies included in this analysis were retrospective in nature. One caveat of our paper was the fact that our analysis did not include an in-depth analysis of patients’ stratification depending on severity and their subsequent outcome. We felt that a dedicated paper reporting these findings was more appropriate because of their ultimate clinical and scientific importance.

## 5. Conclusions

The data in this review provide the most up to date and comprehensive synthesis of the clinical, laboratory, and radiologic characteristics as well as treatment strategies employed in the management of COVID-19. Its novelty is that it correlated several COVID-19 symptoms and diagnostic findings with each other. We showed again the important distinguishing factors of COVID-19 virus infection such as, leukopenia, the age as a risk factor for developing more severe form of the disease, and CRP and its positive correlation with lung lesions. We furthermore provided insight regarding chills as an important symptom that has been overlooked so far. Additional research, however, is indispensable to elucidate viral and host factors in the pathogenesis of various stages of the infection. Early detection, diagnosis and treatment remain the main pillars of COVID-19 management. The clinical, laboratory and radiologic manifestations of the disease are critical in the identification and treatment of the disease. Our manuscript provides additional data that accumulate with other already published papers and will provide “evidence-based hope” on how to manage this unanticipated and overwhelming pandemic and prevent as many fatalities as possible.

## Figures and Tables

**Table 1:** Main characteristics and findings of the previous published meta-analyses.

Authors	Included Studies, *n*	Sample Size	Study Period	Findings	Comment
Rodriguez-Morales AJ et al. (2020) [13]	19	656	Until 23 February 2020	For 656 patients, fever (88.7%, 95% CI 84.5–92.9%), cough (57.6%, 95% CI 40.8–74.4%) and dyspnea (45.6%, 95%CI 10.9–80.4%) were the most prevalent manifestations. Among the patients, 20.3% (95% CI 10.0–30.6%) required ICU, 32.8% presented with ARDS (95% CI 13.7–51.8), 6.2% (95% CI 3.1–9.3) with shock.	Clinical characteristics, laboratory findings, imaging findings and fatal outcome of patients with COVID-19 are summarized in this article—there is no correlations between clinical characteristics, laboratory findings.
Li et al. (2020) [14]	10	1994	December 2019 to February 2020	The main clinical symptoms of COVID-19 patients were fever (88.5%), cough (68.6%), myalgia or fatigue (35.8%), expectoration (28.2%), and dyspnea (21.9%). The results of the laboratory showed that the lymphocytopenia (64.5%), increase in CRP (44.3%), increase in LDH (28.3%), and leukocytopenia (29.4%) were more common.	Clinical characteristics, laboratory findings and fatal outcome or discharge rate of patients with COVID-19 are summarized in this article—there is no correlations between clinical characteristics and laboratory findings
Zhang et al. (2020) [15]	7	4663	Not mentioned (the paper received 13 April 2020)	Patients with elevated CRP levels, lymphopenia, or LDH require proper management and, if necessary, transfer to the ICU.	Laboratory findings are summarized in this article—not mentioning clinical characteristics.
Zeng et al. (2020) [16]	16	3962	Until 20 March 2020	Patients with COVID-19 in the non-severe group had lower levels for CRP, PCT, IL-6, ESR, SAA and serum ferritin compared with those in the severe group.	The paper highlights the association of inflammatory markers with the severity of COVID-19—not mentioning clinical characteristics.
Wang et al. (2020) [17]	49	1667	Until 31 March 2020	The main symptoms of children were fever [48%, 95% CI: 39%, 56%] and cough (39%, 95% CI: 30%, 48%). The lymphocyte count was below normal level in only 15% (95% CI: 8%, 22%) of children which is different from adult patients.	Clinical characteristics and laboratory findings in children were summarized (not for adults)—there is no correlations between clinical characteristics and laboratory findings
Lovato et al. (2020) [18]	5	1556	Until 24 February 2020	Common symptoms were fever (85.6%), cough (68.7%), and fatigue (39.4%). Lymphopenia (77.2%) and leucopenia (30.1%) were common. Critical cases with complications were 9%, ICU admission was required in 7.3%, invasive ventilation in 3.4%, and mortality was 2.4%.	Clinical characteristics, laboratory findings and fatal outcome of patients with COVID-19 are summarized in this article—there is no correlations between clinical characteristics and laboratory findings.
Fu et al. (2020) [19]	43	3600	24 January 2020 to 28 February 2020	Among COVID-19 patients, fever (83.3% [95% CI 78.4–87.7]), cough (60.3% [54.2–66.3]), and fatigue (38.0% [29.8–46.5]) were the most common clinical symptoms. The most common laboratory abnormalities were elevated CRP (68.6% [58.2–78.2]), decreased lymphocyte count (57.4% [44.8–69.5]) and increased LDH (51.6% [31.4–71.6]). The overall estimated proportion of severe cases and CFR was 25.6% (17.4–34.9) and 3.6% (1.1–7.2), respectively.	The paper summarized the symptoms and laboratory findings associated with CFR—there is no correlations between clinical characteristics and laboratory findings.
Li et al. 2020 [20]	12	2445	1 January 2020 to 14 April 2020	Significant differences between the ICU and non-ICU groups for fever, dyspnea, decreased lymphocyte and platelet counts, and increased leukocyte count, CRP, PCT, LDH, aspartate, aminotransferase, alanine aminotransferase, CK, and creatinine levels (*p* < 0.05).	Investigation of clinical characteristics and outcomes of severe cases of COVID-19—there is no correlations between clinical characteristics and laboratory findings.

Abbreviations: No: Number, CI: confidence interval, COVID-19: coronavirus disease 19, ICU: intensive care unit, ARDS: acute respiratory distress syndrome, CRP: C-reactive protein, LDH: lactate dehydrogenase, PCT: procalcitonin, IL: interleukin, ESR: erythrocyte sedimentation rate, SAA: serum amyloid, CFR: case-fatality rate, CK: creatinine kinase.

**Table 2:** Meta-analysis of clinical symptoms, laboratory findings and imaging findings (random-effect model).

Variable	Number of Studies	Mean/Prevalence (%)	95% CI	Number of Patients	$I^{2}$	*p* Value
Clinical Symptoms						
Fever	30	77	0.69–0.85	2628	98.2	<0.0001
Cough	32	60	0.48–0.71	2110	98.6	0
Fatigue/Myalgia	24	31	0.23–0.40	1005	98.1	<0.0001
Dyspnea	19	25	0.20–0.31	533	97.8	<0.0001
Sputum production	16	23	0.15–0.32	624	97.9	<0.0001
Chest tightness	14	17	0.11–0.23	175	95.8	<0.0001
Pharyngalgia	16	13	0.09–0.16	285	78.4	<0.0001
Chill	4	10	0.04–0.16	85	96.7	<0.0001
Headache	15	10	0.06–0.14	276	91.5	<0.0001
Diarrhea	22	6	0.05–0.08	158	56.0	0.0008
Rhinorrhea	12	6	0.04–0.08	120	75.4	<0.0001
Dizziness	4	6	0.02–0.10	19	50.5	0.1086
Nausea/Vomiting	15	5	0.03–0.06	154	83.9	<0.0001
Hemoptysis	4	3	0.004–0.05	22	59.6	0.0593
Laboratory findings						
Increased WBC	12	12	0.08–0.16	192	90.3	<0.0001
Decreased WBC	12	25	0.18–0.32	536	91.4	<0.0001
Decreased lymphocyte	9	63	0.47–0.78	486	96.6	<0.0001
Increased neutrophil	5	31	0.08–0.55	132	98.1	<0.0001
Decreased neutrophil	4	9	0.02–0.17	46	88.7	<0.0001
Increased platelet	3	6	0.02–0.11	22	56.5	0.1003
Decreased platelet	6	16	0.03–0.29	370	96.9	<0.0001
Increased ALT	5	21	0.14–0.27	229	78.4	0.001
Increased AST	5	29	0.17–0.41	277	92.9	<0.0001
Increased D-dimer	5	48	0.15–0.80	418	99.4	<0.0001
Increased creatinine	4	11	0.03–0.20	69	95.2	<0.0001
Increased CRP	9	66	0.51–0.81	573	98.0	<0.0001
Increased creatine kinase	5	32	0.10–0.54	186	98.8	<0.0001
Increased total bilirubin	4	14	0.06–0.22	120	93.3	<0.0001
Increased procalcitonin	6	36	0.16–0.57	214	98.7	<0.0001
Decreased LDH	5	57	0.32–0.83	477	98.6	<0.0001
Decreased albumin	3	61	0.00–1.00	162	99.9	<0.0001
Increased glucose	2	45	0.34–0.57	110	70.9	0.0636
Chest Imaging						
Bilateral infiltration	16	76	0.67–0.85	2157	98.7	<0.0001
Unilateral infiltration	5	20	0.12–0.28	443	80.3	0.0004

Abbreviations: ALT, alanine transaminase; AST, aspartate transaminase; CRP, c-reactive protein; LDH, lactate dehydrogenase; WBC, white blood cell.

**Table 3:** Meta-analysis of treatment (random-effect model).

Variable	Number of Studies	Mean/Prevalence (%)	95% CI	Number of Patients	$I^{2}$	*p* Value
Antiviral agent	10	0.79	0.64–0.95	653	99.5	0
Antibiotic agent	9	0.78	0.62–0.94	1072	99.3	<0.0001
Antifungal agent	2	0.01	0.01–0.04	31	94.1	<0.0001
Corticosteroid	10	0.25	0.16–0.34	407	95.4	<0.0001
Gamma globulin	6	0.25	0.16–0.34	267	91.5	<0.0001
Oxygen inhalation/nasal cannula	8	0.75	0.53–0.97	930	99.4	<0.0001
Non-invasive ventilation	8	0.23	0.16–0.30	211	96.4	<0.0001
RRT	2	0.08	0.00–0.24	18	89.8	0.0017
ECMO	4	0.03	0.00–0.06	17	72.0	0.0133

Abbreviations: ECMO, extracorporeal membrane oxygenation; RRT, renal replacement therapy.

**Table 4:** Correlations among key clinical characteristics and laboratory finding of patents with Coronavirus Disease 2019 (COVID-19).

	Age	Fever	Cough	Fatigue and Myalgia	Dyspnea	Sputum Production	Chest Tightness	Pharyngalgia	Chill	Headache	Lung Abnormality	WBC	Neutrophil	Lymphocyte	Platelet	D-Dimer	CRP
Age	.	−0.267	0.199	0.112	0.764 **	−0.461	0.503	−0.241	−0.963	−0.518	0.413	0.596 *	0.744 **	−0.651 **	0.006	0.796 *	0.165
		0.179	0.320	0.618	0.000	0.097	0.080	0.407	0.173	0.070	0.143	0.015	0.009	0.005	0.986	0.010	0.628
Fever	−0.267	.	−0.427 *	−0.154	−0.261	0.071	0.331	0.245	0.695	0.148	−0.399	−0.121	−0.406	0.419	−0.120	−0.036	−0.508
	0.179		0.021	0.483	0.296	0.801	0.248	0.361	0.305	0.614	0.141	0.643	0.191	0.094	0.711	0.915	0.092
Cough	0.199	−0.427*	.	0.373	0.291	0.674 **	−0.309	0.155	0.689	0.342	0.023	−0.247	0.106	−0.191	−0.155	−0.218	0.389
	0.320	0.021		0.073	0.242	0.004	0.283	0.568	0.311	0.213	0.935	0.324	0.732	0.447	0.612	0.544	0.189
Fatigueand myalgia	0.112	−0.154	0.373	.	0.336	0.557 *	0.358	0.327	0.821	0.476	0.298	−0.402	−0.296	−0.272	−0.457	0.065	0.109
	0.618	0.483	0.073		0.220	0.031	0.253	0.276	0.179	0.073	0.300	0.137	0.377	0.327	0.158	0.858	0.750
Dyspnea	0.764 **	−0.261	0.291	0.336	.	0.413	0.135	−0.272	0.865	0.016	−0.007	0.728 **	0.797 *	−0.700 *	0.301	0.649	0.164
	0.000	0.296	0.242	0.220		0.270	0.729	0.392	0.335	0.964	0.987	0.007	0.010	0.011	0.431	0.163	0.698
Sputum production	−0.461	0.071	0.674 **	0.557 *	0.413	.	0.707	0.268	0.806	0.759 **	0.303	−0.539	−0.261	−0.234	−0.560	−0.502	0.193
	0.097	0.801	0.004	0.031	0.270		0.181	0.521	0.194	0.007	0.365	0.087	0.533	0.489	0.149	0.310	0.678
Chest tightness	0.503	0.331	−0.309	0.358	0.135	0.707	.	0.295	1.000 **	0.236	0.701	0.002	0.606	−0.208	−0.414	0.357	−0.170
	0.080	0.248	0.283	0.253	0.729	0.181		0.408	.	0.764	0.299	0.997	0.202	0.591	0.586	0.432	0.661
Pharyngalgia	−0.241	0.245	0.155	0.327	−0.272	0.268	0.295	.	0.981	0.046	−0.104	−0.441	−0.501	0.395	−0.260	−0.598	−0.079
	0.407	0.361	0.568	0.276	0.392	0.521	0.408		0.126	0.931	0.824	0.175	0.169	0.229	0.574	0.210	0.840
Chill	−0.963	0.695	0.689	0.821	0.865	0.806	1.000 **	0.981	.	0.900	1.000 **	0.419	1.000 **	1.000 **	1.000 **	−1.000 **	1.000 **
	0.173	0.305	0.311	0.179	0.335	0.194	.	0.126		0.287	.	0.725	.	.	.	.	.
Headache	−0.518	0.148	0.342	0.476	0.016	0.759 **	0.236	0.046	0.900	.	0.007	−0.522	−0.588	0.440	−0.160	−0.755	−0.669
	0.070	0.614	0.213	0.073	0.964	0.007	0.764	0.931	0.287		0.985	0.184	0.220	0.275	0.704	0.140	0.331
Lung abnormality	0.413	−0.399	0.023	0.298	−0.007	0.303	0.701	−0.104	1.000 **	0.007	.	0.164	−0.214	0.082	−0.138	−0.445	0.725
	0.143	0.141	0.935	0.300	0.987	0.365	0.299	0.824	.	0.985		0.630	0.611	0.811	0.705	0.270	0.065
WBC	0.596 *	−0.121	−0.247	−0.402	0.728 **	−0.539	0.002	−0.441	0.419	−0.522	0.164	.	0.696 **	−0.098	0.668 **	0.344	0.597*
	0.015	0.643	0.324	0.137	0.007	0.087	0.997	0.175	0.725	0.184	0.630		0.006	0.700	0.009	0.300	0.024
Neutrophil	0.744 **	−0.406	0.106	−0.296	0.797^*^	−0.261	0.606	−0.501	1.000 **	−0.588	−0.214	0.696 **	.	−0.639 *	0.024	0.686	0.141
	0.009	0.191	0.732	0.377	0.010	0.533	0.202	0.169	.	0.220	0.611	0.006		0.019	0.945	0.061	0.679
Lymphocyte	−0.651 **	0.419	−0.191	−0.272	−0.700 *	−0.234	−0.208	0.395	1.000 **	0.440	0.082	−0.098	−0.639 *	.	0.469	−0.546	−0.039
	0.005	0.094	0.447	0.327	0.011	0.489	0.591	0.229	.	0.275	0.811	0.700	0.019		0.106	0.103	0.899
Platelet	0.006	−0.120	−0.155	−0.457	0.301	−0.560	−0.414	−0.260	1.000 **	−0.160	−0.138	0.668 **	0.024	0.469	.	−0.434	0.703 *
	0.986	0.711	0.612	0.158	0.431	0.149	0.586	0.574	.	0.704	0.705	0.009	0.945	0.106		0.283	0.035
D-dimer	0.796 *	−0.036	−0.218	0.065	0.649	−0.502	0.357	−0.598	−1.000 **	−0.755	−0.445	0.344	0.686	−0.546	−0.434	.	−0.259
	0.010	0.915	0.544	0.858	0.163	0.310	0.432	0.210	.	0.140	0.270	0.300	0.061	0.103	0.283		0.501
CRP	0.165	−0.508	0.389	0.109	0.164	0.193	−0.170	−0.079	1.000 **	−0.669	0.725	0.597 *	0.141	−0.039	0.703 *	−0.259	.
	0.628	0.092	0.189	0.750	0.698	0.678	0.661	0.840	.	0.331	0.065	0.024	0.679	0.899	0.035	0.501	

Abbreviations: CRP, c-reactive protein; *n*, number; WBC, white blood cell; ** Correlation is significant at the 0.01 level (2-tailed); * Correlation is significant at the 0.05 level (2-tailed).

## Supplementary Materials

The following are available online at https://www.mdpi.com/1660-4601/17/14/5026/s1, Table S1: Baseline characteristics of included studies.
