# Supplementary material for: Correlations of Clinical and Laboratory Characteristics of COVID-19: A Systematic Review and Meta-Analysis
Source: Int J Environ Res Public Health. 2020 Jul 13;17(14):5026. doi: 10.3390/ijerph17145026 (PMC7400047; doi:10.3390/ijerph17145026)
Supplement: Supplementary file 1 [file ijerph-17-05026-s001.pdf]

Table S1. Baseline characteristics of included studies.

Author, year	Country	Follow-up days	Research type	Number	Reference
Jin-jin Zhang, 2020	China	19	Retrospective study	120	[1]
Xiao-Wei XU,2020	China	16	Retrospective study	62	[2]
Kui Liu, 2020	China	26	Retrospective study	137	[3]
Dawei Wang, 2020	China	34	Retrospective study	138	[4]
Nanshan Chen, 2020	China	25	Retrospective study	99	[5]
Chaolin Huang, 2020	China		Retrospective study	41	[6]
Heshui Shi, 2020	China	35	Retrospective study	81	[7]
Xiaobo Yang, 2020	China	34	Retrospective study	52	[8]
Chang, 2020	China	20	Retrospective study	13	[9]
W.Guan, 2020	China	52	Retrospective study	1099	[10]
Chen L, 2020	China		Retrospective study	29	[11]
Jie Li, 2020	China		Retrospective study	17	[12]
Wu WS, 2020	China		Retrospective study	40	[13]
Wei Liu, 2020	China		Retrospective study	83	[14]
Kunhua Li, 2020	China		Retrospective study	83	[15]
Cheng JL, 2020	China		Retrospective study	1079	[16]
Huijun Chen, 2020	China		Retrospective study	9	[17]
Jiong Wu, 2020	China		Retrospective study	80	[18]
Peng Yudong, 2020	China	26	Retrospective study	112	[19]
Jian Wu, 2020	China	23	Retrospective study	80	[20]
Barnaby Edward Young, 2020	Singapore		Retrospective study	18	[21]
Xi Xu, 2020	China	12	Retrospective study	90	[22]
Yao Na, 2020	China		Retrospective study	40	[23]
Sijia Tian, 2020	China		Retrospective study	262	[24]
Bicheng Zhang, 2020	China		Retrospective study	82	[25]
Anjue Tang, 2020	China	23	Retrospective study	26	[26]

Yan Bai, 2020	China		Retrospective study	6	[27]
Adam Bernheim, 2020	China	15	Retrospective study	121	[28]
wenjie yang, 2020	China		Retrospective study	149	[29]
fengxiang song, 2020	China		Retrospective study	51	[30]
Feng K, 2020	China	21	Retrospective study	15	[31]
Li YY, 2020	China		Retrospective study	31	[32]
Michael Chung, 2020	China	9	Retrospective study	21	[33]
Feng Pan, 2020	China	25	Retrospective study	21	[34]
Matt Arentz, 2020	Washington, USA	15	Retrospective study	21	[35]
Yinxiaohe Sun, 2020	Singapore	21	Retrospective study	788	[36]
Summary	-	0-52	Retrospective study	5,196	

References

1. Zhang, J.-J.; Dong, X.; Cao, Y.-Y.; Yuan, Y.-D.; Yang, Y.-B.; Yan, Y.-Q.; Akdis, C.A.; Gao, Y.-D. Clinical characteristics of 140 patients infected with SARS-CoV-2 in Wuhan, China. *Allergy* **2020**, doi:10.1111/all.14238.
2. Xu, X.-W.; Wu, X.-X.; Jiang, X.-G.; Xu, K.-J.; Ying, L.-J.; Ma, C.-L.; Li, S.-B.; Wang, H.-Y.; Zhang, S.; Gao, H.-N.; et al. Clinical findings in a group of patients infected with the 2019 novel coronavirus (SARS-Cov-2) outside of Wuhan, China: Retrospective case series. *BMJ* **2020**, 368, m606.
3. Liu, K.; Fang, Y.-Y.; Deng, Y.; Liu, W.; Wang, M.-F.; Ma, J.-P.; Xiao, W.; Wang, Y.-N.; Zhong, M.-H.; Li, C.-H.; et al. Clinical characteristics of novel coronavirus cases in tertiary hospitals in Hubei Province. *Chin. Med J.* **2020**, *133*, 1025–1031, doi:10.1097/cm9.0000000000000744.
4. Wang, D.; Hu, B.; Hu, C.; Zhu, F.; Liu, X.; Zhang, J.; Wang, B.; Xiang, H.; Cheng, Z.; Xiong, Y.; et al. Clinical Characteristics of 138 Hospitalized Patients With 2019 Novel Coronavirus–Infected Pneumonia in Wuhan, China. *JAMA* **2020**, *323*, 1061, doi:10.1001/jama.2020.1585.
5. Chen, N.; Zhou, M.; Dong, X.; Qu, J.; Gong, F.; Han, Y.; Qiu, Y.; Wang, J.; Liu, Y.; Wei, Y.; et al. Epidemiological and Clinical Characteristics of 99 Cases of 2019-Novel Coronavirus (2019-nCoV) Pneumonia in Wuhan, China. *SSRN Electron. J.* **2020**, *395*, 507–513, doi:10.2139/ssrn.3523861.
6. Huang, C.; Wang, Y.; Li, X.; Ren, L.; Zhao, J.; Hu, Y.; Zhang, L.; Fan, G.; Xu, J.; Gu, X.; et al. Clinical features of patients infected with 2019 novel coronavirus in Wuhan, China. *Lancet* **2020**, *395*, 497–506.
7. Shi, H.; Han, X.; Jiang, N.; Cao, Y.; Alwalid, O.; Gu, J.; Fan, Y.; Zheng, C. Radiological findings from 81 patients with COVID-19 pneumonia in Wuhan, China: a descriptive study. *Lancet Infect. Dis.* **2020**, *20*, 425–434, doi:10.1016/s1473-3099(20)30086-4.
8. Yang, X.; Yu, Y.; Xu, J.; Shu, H.; Xia, J.; Liu, H.; Wu, Y.; Zhang, L.; Yu, Z.; Fang, M.; et al. Clinical course and outcomes of critically ill patients with SARS-CoV-2 pneumonia in Wuhan, China: a single-centered, retrospective, observational study. *Lancet Respir. Med.* **2020**, *8*, 475–481, doi:10.1016/s2213-2600(20)30079-5.
9. Chang, D.; Lin, M.; Wei, L.; Xie, L.; Zhu, G.; Cruz, C.S.D.; Sharma, L. Epidemiologic and Clinical Characteristics of Novel Coronavirus Infections Involving 13 Patients Outside Wuhan, China. *JAMA* **2020**, *323*, 1092, doi:10.1001/jama.2020.1623.
10. Guan, W.-J.; Ni, Z.-Y.; Hu, Y.; Liang, W.-H.; Ou, C.-Q.; He, J.-X.; Liu, L.; Shan, H.; Lei, C.-L.; Hui, D.S.; et al. Clinical Characteristics of Coronavirus Disease 2019 in China. *New Engl. J. Med.* **2020**, *382*, 1708–1720, doi:10.1056/nejmoa2002032.

11. Chen, L.; Liu, H.G.; Liu, W.; Liu, J.; Liu, K.; Shang, J.; Deng, Y.; Wei, S. [Analysis of clinical features of 29 patients with 2019 novel coronavirus pneumonia]. Zhonghua Jie He He Hu Xi Za Zhi **2020**, *43*, 203–208.
12. Li, J.; Li, S.; Cai, Y.; Liu, Q.; Li, X.; Zeng, Z.; Chu, Y.; Zhu, F.; Zeng, F. Epidemiological and Clinical Characteristics of 17 Hospitalized Patients with 2019 Novel Coronavirus Infections Outside Wuhan, China 2020.
13. Wu, W.S.; Li, Y.G.; Wei, Z.F.; Zhou, P.H.; Lyu, L.K.; Zhang, G.P.; Zhao, Y.; He, H.Y.; Li, X.Y.; Gao, L.; et al. [Investigation and analysis on characteristics of a cluster of COVID-19 associated with exposure in a department store in Tianjin]. *Zhonghua Liu Xing Bing Xue Za Zhi* **2020**, *41*, 489–493.
14. Liu, W.; Tao, Z.-W.; Wang, L.; Yuan, M.-L.; Liu, K.; Zhou, L.; Wei, S.; Deng, Y.; Liu, J.; Liu, H.-G.; et al. Analysis of factors associated with disease outcomes in hospitalized patients with 2019 novel coronavirus disease. *Chin. Med J.* **2020**, *133*, 1032–1038, doi:10.1097/cm9.0000000000000775.
15. Li, K.; Wu, J.; Wu, F.; Guo, D.; Chen, L.; Fang, Z.; Li, C. The Clinical and Chest CT Features Associated With Severe and Critical COVID-19 Pneumonia. *Investig. Radiol.* **2020**, *55*, 327–331, doi:10.1097/rli.0000000000000672.
16. Cheng, J.L.; Huang, C.; Zhang, G.J.; Liu, D.W.; Li, P.; Lu, C.Y.; Li, J. [Epidemiological characteristics of novel coronavirus pneumonia in Henan]. *Zhonghua Jie He He Hu Xi Za Zhi 2020* 2020, *43*, E027.
17. Chen, H.; Guo, J.; Wang, C.; Luo, F.; Yu, X.; Zhang, W.; Li, J.; Zhao, D.; Xu, D.; Gong, Q.; et al. Clinical characteristics and intrauterine vertical transmission potential of COVID-19 infection in nine pregnant women: a retrospective review of medical records. *Lancet* **2020**, *395*, 809–815, doi:10.1016/s0140-6736(20)30360-3.
18. Wu, J.; Wu, X.; Zeng, W.; Guo, D.; Fang, Z.; Chen, L.; Huang, H.; Li, C. Chest CT Findings in Patients With Coronavirus Disease 2019 and Its Relationship With Clinical Features. *Investig. Radiol.* **2020**, *55*, 257–261, doi:10.1097/rli.0000000000000670.
19. Peng, Y.D.; Meng, K.; Guan, H.Q.; Leng, L.; Zhu, R.R.; Wang, B.Y.; A He, M.; Cheng, L.X.; Huang, K.; Zeng, Q.T. [Clinical characteristics and outcomes of 112 cardiovascular disease patients infected by 2019-nCoV] 2020, *48*, E004.
20. Wu, J.; Liu, J.; Zhao, X.; Liu, C.; Wang, W.; Wang, D.; Xu, W.; Zhang, C.; Yu, J.; Jiang, B.; et al. Clinical Characteristics of Imported Cases of Coronavirus Disease 2019 (COVID-19) in Jiangsu Province: A Multicenter Descriptive Study. *Clin. Infect. Dis.* **2020**, doi:10.1093/cid/ciaa199.
21. Young, B.E.; Ong, S.W.X.; Kalimuddin, S.; Low, J.G.; Tan, S.Y.; Loh, J.; Ng, O.-T.; Marimuthu, K.; Ang, L.W.; Mak, T.M.; et al. Epidemiologic Features and Clinical Course of Patients Infected With SARS-CoV-2 in Singapore. *JAMA* **2020**, *323*, 1488–1494, doi:10.1001/jama.2020.3204.
22. Xu, X.; Yu, C.; Qu, J.; Zhang, L.; Jiang, S.; Huang, D.; Chen, B.; Zhang, Z.; Guan, W.; Ling, Z.; et al. Imaging and clinical features of patients with 2019 novel coronavirus SARS-CoV-2. *Eur. J. Nucl. Med. Mol. Imaging* **2020**, *47*, 1275–1280, doi:10.1007/s00259-020-04735-9.
23. Yao, N.; Wang, S.N.; Lian, J.Q.; Sun, Y.T.; Zhang, G.F.; Kang, W.Z.; Kang, W. [Clinical characteristics and influencing factors of patients with novel coronavirus pneumonia combined with liver injury in Shaanxi region] 2020, *28*, E003.
24. Tian, S.; Hu, N.; Lou, J.; Chen, K.; Kang, X.; Xiang, Z.; Chen, H.; Wang, D.; Liu, N.; Liu, D.; et al. Characteristics of COVID-19 infection in Beijing. *J. Infect.* **2020**, *80*, 401–406.
25. Zhang, B.; Zhou, X.; Qiu, Y.; Feng, F.; Feng, J.; Jia, Y.; Zhu, H.; Hu, K.; Liu, J.; Liu, Z.; et al. Clinical characteristics of 82 death cases with COVID-19 2020.
26. Tang, A.; Xu, W.; Shen, M.; Chen, P.; Li, G.; Liu, Y.; Liu, L. A retrospective study of the clinical characteristics of COVID-19 infection in 26 children 2020.
27. Bai, Y.; Yao, L.; Wei, T.; Tian, F.; Jin, D.-Y.; Chen, L.; Wang, M. Presumed Asymptomatic Carrier Transmission of COVID-19. *JAMA* **2020**, *323*, 1406, doi:10.1001/jama.2020.2565.
28. Bernheim, A.; Mei, X.; Huang, M.; Yang, Y.; Fayad, Z.; Zhang, N.; Diao, K.; Lin, B.; Zhu, X.; Li, K.; et al. Chest CT Findings in Coronavirus Disease-19 (COVID-19): Relationship to Duration of Infection. *Radiol.* **2020**, *295*, 2020200463, doi:10.1148/radiol.2020200463.
29. Yang, W.; Cao, Q.; Qin, L.; Wang, X.; Cheng, Z.; Pan, A.; Dai, J.; Sun, Q.; Zhao, F.; Qu, J.; et al. Clinical characteristics and imaging manifestations of the 2019 novel coronavirus disease (COVID-19):A multi-center study in Wenzhou city, Zhejiang, China. *J. Infect.* **2020**, *80*, 388–393, doi:10.1016/j.jinf.2020.02.016.
30. Song, F.; Shi, N.; Shan, F.; Zhang, Z.; Shen, J.; Lu, H.; Ling, Y.; Jiang, Y.; Shi, Y. Emerging 2019 Novel Coronavirus (2019-nCoV) Pneumonia. *Radiol.* **2020**, *295*, 210–217, doi:10.1148/radiol.2020200274.

31. Feng, K.; Yun, Y.X.; Wang, X.F.; Yang, G.D.; Zheng, Y.J.; Lin, C.M.; et al. Analysis of CT features of 15 Children with 2019 novel coronavirus infection. *Zhonghua Er Ke Za Zhi* **2020**, *58*, E007.
32. Li, Y.Y.; Wang, W.N.; Lei, Y.; Zhang, B.; Yang, J.; Hu, J.W.; Ren, Y.L.; Lu, Q.F. [Comparison of the clinical characteristics between RNA positive and negative patients clinically diagnosed with 2019 novel coronavirus pneumonia] 2020, *43*, E023.
33. Chung, M.; Bernheim, A.; Mei, X.; Zhang, N.; Huang, M.; Zeng, X.; Cui, J.; Xu, W.; Yang, Y.; Fayad, Z.A.; et al. CT Imaging Features of 2019 Novel Coronavirus (2019-nCoV). *Radiol.* **2020**, *295*, 202–207, doi:10.1148/radiol.2020200230.
34. Pan, F.; Ye, T.; Sun, P.; Gui, S.; Liang, B.; Li, L.; Zheng, D.; Wang, J.; Hesketh, R.L.; Yang, L.; et al. Time Course of Lung Changes at Chest CT during Recovery from Coronavirus Disease 2019 (COVID-19). *Radiol.* **2020**, *295*, 715–721, doi:10.1148/radiol.2020200370.
35. Arentz, M.; Yim, E.; Klaff, L.; Lokhandwala, S.; Riedo, F.X.; Chong, M.; Lee, M. Characteristics and Outcomes of 21 Critically Ill Patients With COVID-19 in Washington State. *JAMA* **2020**, *323*, 1612, doi:10.1001/jama.2020.4326.
36. Sun, Y.; Koh, V.; Marimuthu, K.; Ng, O.T.; Young, B.; Vasoo, S.; Chan, M.; Lee, V.J.M.; De, P.P.; Barkham, T.; et al. Epidemiological and Clinical Predictors of COVID-19. *Clin. Infect. Dis.* **2020**, doi:10.1093/cid/ciaa322.
